# β-3 adrenergic receptor gene polymorphisms are associated with gestational diabetes mellitus in a Chinese population

**DOI:** 10.1097/MD.0000000000017258

**Published:** 2019-10-25

**Authors:** He Jia, Yuan Pan, Yun Wang, Feng-Ling Yin, Na Xu

**Affiliations:** a Department of Obstetrics and Gynecology, the 940th Hospital of Lianqin Security Force, Lanzhou; b Center for Reproductive Medicine and Center for Prenatal Diagnosis, First Hospital, Jilin University, Changchun, 130021, China; c Department of Obstetrics and Gynecology, Xuzhou central hospital, Xuzhou; d Department of Obstetrics, Wuxi People's Hospital Affiliated to Nanjing Medical University, Wuxi, China.

**Keywords:** β-3 adrenergic receptor, gestational diabetes mellitus, rs201607471, single nucleotide polymorphism

## Abstract

Increasing studies demonstrated that genetic susceptibility attributes to the development of gestational diabetes mellitus (GDM). The polymorphisms of the β-3 adrenergic receptor(β-3AR) gene have been found to be of great importance in bodyweight elevation and dyslipidaemias. We aimed to determine the influence of β-3AR polymorphisms on the GDM risk. Thus, we performed a case-control study including 136 GDM cases and 138 controls to evaluate the relation between the rs201607471 and susceptibility to GDM. Likelihood ratios *X*^*2*^ analysis showed the distribution of the genotype frequency (rs201607471 in β-3AR gene) was accorded with the Hardy-Weinberg genetic equilibrium. Although no significant association between rs201607471 alleles and GDM susceptibility (Chi-square test, *P* > .05), we observed that β-3AR gene rs201607471 *CT* genotype was significantly prevalent in GDM (Chi-square test, *P* < .05). Moreover, we observed that β-3AR gene rs201607471 *C* > T was significantly associated with an increased risk of GDM using the recessive model (CC vs CT/TT: *P* = .026) and the additive model (CC vs CT vs TT: *P* = .038). These data indicate that β-3AR rs201607471 may be a helpful susceptibility marker for GDM in Chinese pregnant women.

## 1 Introduction

Gestational diabetes mellitus (GDM) is a common metabolic disorder syndrome during pregnancy.^[1]^ GDM can affect the health of pregnant women and fetuses to various extent, increasing the risk of other obstetric complications, followed by a higher incidence of type 2 diabetes and cardiovascular disease within 10 years after delivery.^[2,3]^ The symptoms of GDM are concealed, with the lack of typical diabetes manifestations in pregnant women, resulting in treatment delay with unimaginable consequences. Early prediction and prevention of risk factors have a vital role in GDM related crisis.

Kim et al^[4]^ showed that the cumulative incidence of diabetes could reach 70% among the examined women in 11 years postpartum, which could be aggravated by a family history of diabetes. It is the prevalence of GDM not only attribute to acquired environmental factors but also congenital genetic factors.^[5–7]^ If genetics such as genetic polymorphism can be used to predict the risk of GDM, the disease and its crisis will be effectively prevented.

The β-3 adrenergic receptor (β-3AR), a protein polypeptide consisting of 402 amino acids, is newly discovered after β-1 and β-2. It is a member of the G protein-coupled receptor superfamily, mainly mediates the decomposition and heat generation of fat.^[8]^ Insulin resistance, which could be triggered by β-3AR gene mutation,^[9]^ is a major risk factor for GDM. Therefore, we enrolled the GDM pregnant women in our hospital, evaluated the polymorphism of rs201607471 in β-3AR gene by TaqMan-MGB probe method, and explored the relevance between β-3AR gene polymorphism and the pathogenesis of GDM.

## 2 Materials and methods

### 2.1 Population

GDM patients (n = 136) and healthy pregnant women (n = 138) were enrolled in the Department of Obstetrics, Wuxi People's Hospital, Nanjing Medical University, from January 2016 to January 2018. We retrospectively collected baseline data of pregnant women from the registration system in Wuxi People Hospital, including age, health data before pregnancy (body mass index, weight, height, and hypertension or not), Family history of diabetes, pregnancy history (with GDM or macrosomia). We attained corresponding blood remnants from the Department of Clinical Laboratory just after the first routine check in our hospital. Considering the reasons mentioned above, the Ethics Committee of Wuxi People Hospital exempted this study from requiring ethics approval.

GDM was diagnosis using the 2011 American Diabetes Association (ADA) diagnostic criteria: Pregnant women at 24 to 28 weeks, 75 g oral glucose tolerance test (OGTT), fasting blood glucose≥5.1mmol/L, 1 hour blood glucose≥10.0mmol/L, 2 hours blood glucose ≥8.5 mmol/L, blood glucose at any time to meet or exceed the above criteria can be diagnosed as GDM; Compliance is good and the information is complete. Exclusion criteria: Patients with abnormal glucose metabolism before pregnancy; Combined with other pregnancy complications including intrahepatic cholestasis during pregnancy, hypertensive disorder complicating pregnancy, thyroid dysfunction; Combined with mental illness or other cognitive dysfunction cannot Cooperating patients; Heart, kidney, liver and the other important organ dysfunction. According to the above criteria, 136 patients with GDM were included in the study as the experimental group, 138 healthy pregnant women with no abnormalities in the hospital were selected as the control group. All subjects were unrelated Han Chinese. Baseline data of the study population were collected, including the name, age, weight, height, and body mass index (BMI).

### 2.2 Measurement of plasma indicator

Fasting insulin (FINS), fasting plasma glucose (FPG), glucose 1 hours after OGTT, glucose 2 hours after OGTT, cholesterol (CHO), triglyceride (TG), low-density lipoprotein (LDL-C), and High-density lipoprotein (HDL-C) levels were retrospectively attained from the registration system of Wuxi People's Hospital. All markers were investigated using an automatic biochemical analyzer (Beckman, USA) in the Department of Clinical Laboratory, Wuxi People's Hospital, Nanjing Medical University.

### 2.3 Blood samples and SNP analysis

The 1 mL blood of the study subject was harvested from the Department of Clinical Laboratory, Wuxi People's Hospital, Nanjing Medical University. The DNA was extracted using the medium-quantity whole blood genomic DNA extraction kit (Beijing Biotech Biotechnology Co., Ltd.) The specific extraction procedure was carried out according to the manufacturer's instructions. The genotype detection analysis of the sample was performed using TaqMan SNP Genotyping Assays kit (Thermo Fisher Scientific, USA). Specific gene probe information is shown in Table 1.

**Table 1:**
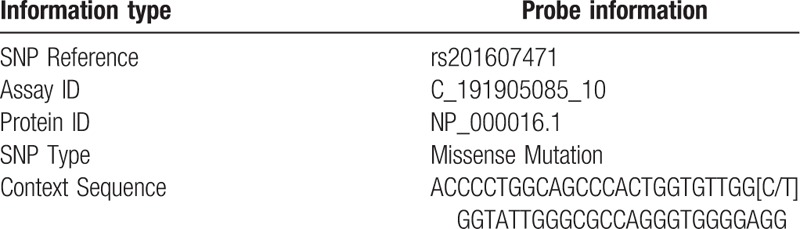
Probe information for rs201607471 in β-3AR gene.

### 2.4 Statistical Methods

Statistical analysis was performed by SPSS 19.0 statistical software. The results of the measurement data were expressed as mean ± SD, and the comparison between the 2 groups of measurement data was performed using an independent Student's *t* test. Whether the distribution of each genotype was consistent with the Hardy–Weinberg equilibrium law was evaluated by the means of the likelihood ratio *X*^*2*^ test. The Chi-square test was used to compare the genotype and allele frequencies of each group. The difference was statistically significant as *P* < .05.

## 3 Result

### 3.1 Patient characteristics

The characteristics of study pregnant women are shown in Table 2. A total of 274 women enrolled in our retrospective study. Of the total participants, 136 women (49.6%) had a diagnosis of GDM with an average age of 31.94 ± 5.86 years, while 138 pregnant women (50.4%) were in comprehensive health condition with an average age of 27.38 ± 3.74 years. As shown in Table 2, BMI and weight were higher in GDM patients, who compared with healthy pregnant women (all *P* < .05). Lower fasting insulin, worse OGTT performance, and higher LDL-C appeared in GDM patients, but not healthy pregnant women (all *P* < .01).

**Table 2:**
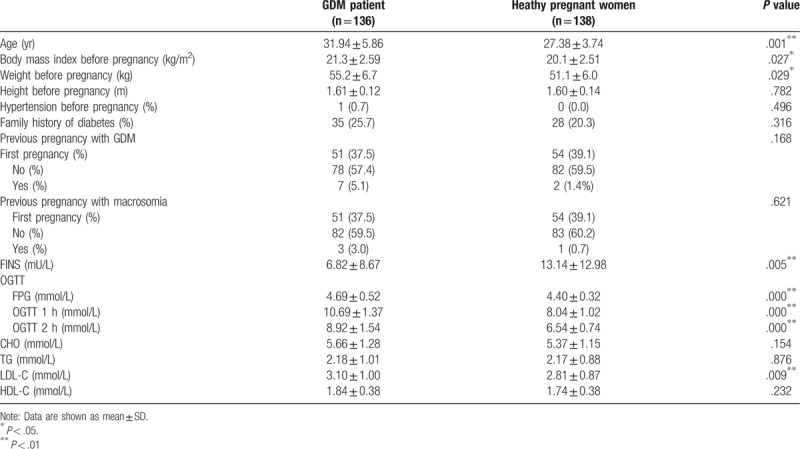
Characteristics of the study population.

### 3.2 CT genotype seems to be associated with a high risk of GDM

Before we evaluate the relevancy between GDM and the polymorphism of β-3AR gene at rs201607471, we assessed the consistency of frequency with Hardy–Weinberg law of genetic equilibrium. Comparing the frequency from three genotypes between 2 observed groups using the likelihood ratios *X*^*2*^ test. The results showed that both the distribution of the genotype frequency (rs201607471 in β-3AR gene) in these 2 groups were accorded with the Hardy-Weinberg genetic equilibrium (*X*^*2*^ = 0.010, *P* = .99) (Table 3). The frequencies of *C* and *T* alleles in the patient group were 77.57% and 22.43%, respectively, while the ones in the healthy group were 83.33% and 16.67%. However, statistics demonstrated no difference in allele distribution between these two groups (*P* > .05) (Table 4). Moreover, analysis of genotypes frequencies showed that *CT* inclined distinctively in the patient group with 41.91%, but not *CC* (56.62%) nor *TT* (1.47%), compared to 27.54% (*P* < .05) in the healthy group with 69.57% *CC* and 2.89% *TT* genotype (Table 5). These results suggested that *CT* genotype may be related to the high risk of GDM

**Table 3:**
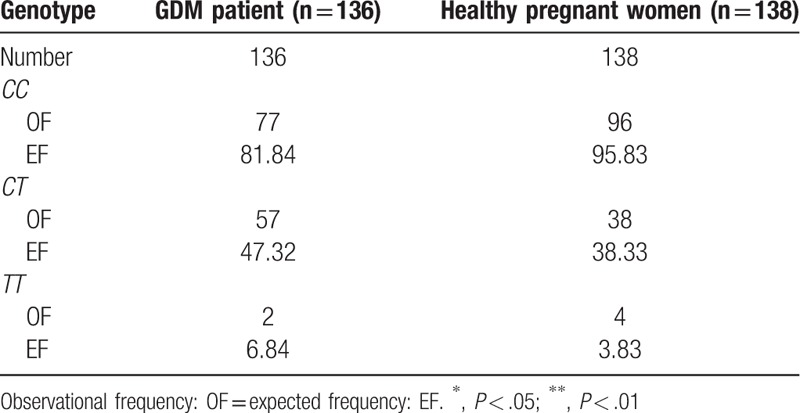
Genetic equilibrium of the study population.

**Table 4:**
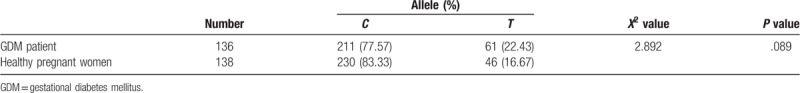
Comparison of the distribution of rs201607471C/T alleles in β-3AR gene between 2 groups.

**Table 5:**
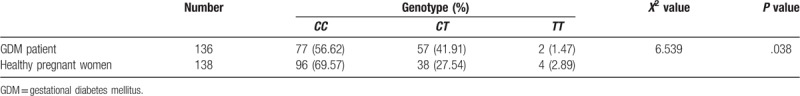
Comparison of the distribution of rs201607471 genotype in β-3AR gene between 2 groups.

### 3.3 The recessive genotype is more likely relative to a high risk of GDM

Considering the observational number of *TT* genotype is quite low in both groups as shown in Table 5, we further investigated the genetic model difference between these two groups. The recessive model was more likely the main genetic model of rs201607471(C/T) in GDM patients (*P* < .05), compared with healthy pregnant women (*P* > .05) (Table 6). These data indicated that TT genotype in β-3AR gene rs201607471represented the susceptibility of GDM among pregnant women.

**Table 6:**
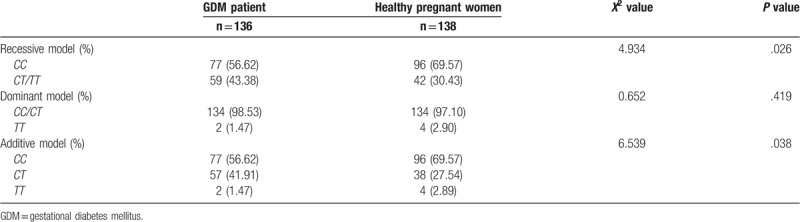
The genetic model distribution of rs201607471 in β-3AR gene between 2 groups.

## 4 Discussion

The secretion of hormone (estrogen, progesterone, prolactin and placental prolactin) and cytokines antagonist to insulin is highly activated during pregnancy, whereas the demand for insulin remains increasing in pregnant women. Insufficient supplement of insulin will lead to the initiation of GDM.^[10]^ Rising studies proposed the vital function of the genetic susceptibility in GDM. *GG* genotype and *G* allele of SNP-420C>G aggravated the risk of GDM in the Iranian population.^[11]^ Rs10830963/G of MTNR1B represented the most important risk alleles associated with GDM.^[12]^ The polymorphisms of the β-3AR gene proposed in our study, have been proved to play a significant role in bodyweight elevation and dyslipidaemias.^[13,14]^ Investigator found that, in all genetic models with random effects except HTG model, it exhibited significant associations between type 2 diabetes and rs4994 polymorphism of the β-3AR gene in Asians.^[15]^ However, the non-Asian population demonstrated no significant association in any genetic models with random effects.^[15]^ The role of the β-3AR gene in GDM has been controversial. A search in Roma for beta3-AR gene polymorphism (ADRB3 Trp64Arg) showed no significant differences between women with GDM and women with normal glucose tolerance.^[16]^ A meta-analysis was conducted on associations between Trp64Arg polymorphism in ADRB3 and susceptibility to GDM. Another meta-analysis addressed that no association between Trp64Arg polymorphism in ADRB3 and susceptibility to GDM was found in overall population (Arg vs Trp: OR = 1.20, 95%CI = 0.99–1.47, *P* = .16; Trp/Arg+Arg/Arg vs Trp/Trp: OR = 1.22, 95%CI = 0.99–1.50, *P* = .11).^[17]^ However, Trp64Arg polymorphism in ADRB3 had a certain association with susceptibility to GDM in subgroup analysis on European Caucasian.^[17]^

In this study, we analyzed the genotype and allele regarding the β-3AR polymorphism locus rs201607471 (C/T) between GDM patients and the control women via TaqMan-MGB probe method. As far as know, our original research is the first report on the impact of the locus rs201607471 (C/T) at the β-3AR gene on the development of GDM. Although the distribution of *C* and *T* alleles between the 2 groups *C* or *T* seems similar (Table 4), the frequency of *CT* genotype in rs201607471 is higher in GDM patients (Table 5), suggesting that the β-3AR gene rs201607471 (C/T) polymorphism is associated with the risk of GDM. Further, the recessive mode is the significant genetic model of rs201607471(C/T) in GDM patients, compared with healthy pregnant women gene. Instead of the result shown in Table 5, genetic model analysis in Table 6 more likely takes small cases to bias into account in attribution analysis, showing that recessive mode appears more frequently, which indicated that *TT* suitable for describing the inheritance of β-3AR gene rs201607471 in GDM.

The β-3 adrenergic receptor plays an important role in the initiation of lipid decomposition, storage, and utilization of energy, and internal environmental stability of glucose.^[18]^ Mutations in the β-3AR gene lead to defects in gene expression of surface receptors on the cell membrane, followed by damage of signals transduction, which will result in lipolysis of intracellular adipose tissue.^[3,19–21]^ Those are believed as the initiation of insulin resistance, diabetes, obesity, etc. However, certainly, this cannot fully clarify the pathophysiology for GDM due to the significant role of interaction between genetics and the environment. Further investigation should be carried out to provide additional insight into the gene-by-environment interaction.

Therefore, by the means of the comparisons of the baseline information and serum biochemical markers between GDM pregnant women and healthy pregnant women, our study found that the age, BMI and weight were more likely high in GDM women, whose FINS was lower, however (Table 2). Besides, FPG, OGTT 1 h, OGTT 2 h, and LDL-C were aberrantly high compared with healthy pregnant women. All these indicated that a series of factors including advanced age, obesity, decreased islet function, impaired glucose tolerance, and dyslipidemia increase the risk of GDM, which is consistent with previous reports.^[22–24]^ Overweight and obese pregnant women are often accompanied by hyperlipidemia, etc., all of which can aggravate the severity of insulin resistance and increase the incidence of GDM.^[25,26]^ The lower the FINS, the worse hypoglycemic activity Insulin cells have, which leads to abnormal OGTT. Therefore, during the pregnancy care process, more attention should be paid to blood sugar and BMI from pregnant women, especially the elderly ones.

## Author contributions

**Data curation:** He Jia.

**Formal analysis:** He Jia.

**Funding acquisition:** Na Xu.

**Investigation:** He Jia.

**Methodology:** Yuan Pan.

**Project administration:** Na Xu.

**Resources:** Fengling Yin.

**Software:** Yuan Pan.

**Validation:** Yuan Pan, Yun Wang.

**Visualization:** Yun Wang, Fengling Yin.

**Writing – original draft:** Yuan Pan.

**Writing – review & editing:** Na Xu.
